# Anatomical variations of the frontal sinus: A computed tomography-based study

**DOI:** 10.12688/f1000research.129498.2

**Published:** 2023-08-31

**Authors:** Asma Sulaiman Al Hatmi, Eiman Al Ajmi, Halima Albalushi, Meetham Al Lawati, Srinivasa Rao Sirasanagandla

**Affiliations:** 1 Department of Radiology, Ibri Hospital, Ibri, 512, Oman; 2 Department of Radiology and Molecular Imaging, College of Medicine and Health Sciences, Sultan Qaboos University, Al Khoudh, Muscat, 123, Oman; 3 Department of Human and Clinical Anatomy, College of Medicine and Health Sciences, Sultan Qaboos University, Al khoudh, Muscat, 123, Oman; 4 College of Medicine and Health Sciences, Sultan Qaboos University, Al Khoudh, Muscat, 123, Oman

**Keywords:** Frontal sinus, sinusitis, hyperplasia, aplasia, surgery

## Abstract

**Background:** The pneumatization of the frontal sinus is variable between individuals, including monozygotic twins. The volumetric anatomic variants of the frontal sinus are classified into aplasia, hypoplasia, medium-sized, and hyperplasia. We aimed to study the frontal sinus morphology in Omani patients using computed tomography (CT) evaluations.

**Methods:** Retrospectively, 1220 frontal sinus CT scans from 610 patients investigated at Sultan Qaboos University Hospital, Oman, from January 2019 to December 2020 were reviewed. The frontal sinus morphology was classified according to the classification proposed by Guerram *et al.* The Chi-square test was used to determine the influence of sex.

**Results:** With regard to the unilateral occurrence, the most prevalent frontal sinus category observed was medium-sized (13.3%), followed by hyperplasia (7.9%), hypoplasia (5.4%), and aplasia (2%) categories. Similarly, in bilateral occurrence, the most common frontal sinus category observed was medium-sized (53%), followed by hyperplasia (13.1%), hypoplasia (3.4%) and aplasia (2%) categories. Right and left frontal sinus aplasia were observed in 2.1% and 1.8% of cases, respectively. In terms of sex influence, the left unilateral ( *p*<0.01) and the bilateral hypoplasia ( *p*<0.05) were significantly higher in females. On the other hand, the left unilateral ( *p*<0.01) and the bilateral hyperplasia ( *p*<0.05) were higher in males.

**Conclusions:** The baseline data of frontal sinus category frequencies reported in the present study is helpful in the diagnostic evaluation of sinusitis in the clinical setting. The preoperative recognition of frontal sinus types, particularly frontal sinus aplasia in multiplanar CT scans, is crucial to avoid unexpected complications while performing endoscopic sinus surgery.

## Introduction

Frontal sinuses are a pair of funnel-shaped pneumatic cavities situated in the squamous part of the temporal bone. Two frontal sinuses are separated by a bony septum, which is rarely located in the midline. ^1^ Frontal sinuses emerge as an outgrowth in the region of the frontal recess of the nose in the fourth month of intrauterine life. At birth, they are rudimentary or absent. They begin to develop and become evident only after the second year of life. They grow at the age of seven or eight and mature after puberty. ^2^^,^^3^ After 20 years of age, the size of the sinuses remains unchanged until the atrophic changes begin to appear due to advancing age. ^4^ Frontal sinuses drain into the anterior part of the middle meatus of the nose through an ethmoid infundibulum or a frontonasal duct. The volume of the frontal sinus is highly variable between the two individuals. In adults, the mean size of the sinus is around ten cc and it may reach a maximum of 37 cc. ^5^

Frontal sinuses are rarely symmetrical as both sinuses develop individually. ^5^ The pneumatization of the frontal sinus is known to be highly variable. The frontal sinus morphology varies from aplasia to hyperplasia within the same individual and even between monozygotic twins. ^6^ The anatomical variations of the frontal sinus morphology have been reported in various populations worldwide. ^1^^,^^7^^–^^12^ These studies have confirmed that climate and geography influence the frontal sinus morphology differences between the populations. In addition, few studies have demonstrated sexual dimorphism in frontal sinus anatomic variations. ^6^^,^^13^^–^^15^ Frontal sinus anatomic variations are clinically important as they are closely associated with frontal sinusitis physiopathology, clinical presentation, development of complications and treatment. ^16^^–^^20^ Furthermore, these variations and the unique morphology of the frontal sinus are helpful in the identification of subjects for forensics. ^14^^,^^21^ Previously, few studies have proposed classifications for frontal sinus morphology based on two-dimensional and three-dimensional evaluations. Despite tremendous clinical significance, few recent papers have dealt with frontal sinus morphology, particularly in Middle Eastern populations. Hence, the objective of the present study was to assess the frontal sinus morphology in Omani patients using computed tomography (CT) according to the classification by Guerram *et al.* ^10^

## Methods

### Patient population

The present study is a retrospective review of the electronic medical records database (TrakCare Unified Health Information System) at the Department of Radiology and Molecular Imaging, Sultan Qaboos University Hospital, Oman. All Omani patients aged ≥18 years referred for CT scan of the paranasal sinuses from January 2019 to December 2020 were included in the study. Patients with anterior skull base trauma, fibro-osseous lesions or significant motion artifacts that impaired the visualization of the frontal sinuses were excluded from the study. The present study obtained institutional ethical approval from the Medical Research Ethics Committee, Sultan Qaboos University.

### CT acquisition protocol

All the scans were performed using a 64 multidetector CT scanner (Siemens Sensation 64) with the following parameters: 120 kVp, tube current modulation with reference mAs of 130 and 0.75-mm slice thickness. The Picture Archiving and Communication System (PACS) (Synapse PACS, FUJIFILM Worldwide, version 5.7.102) was used for screening the scans.

### Data collection and definitions

**Figure 1.**
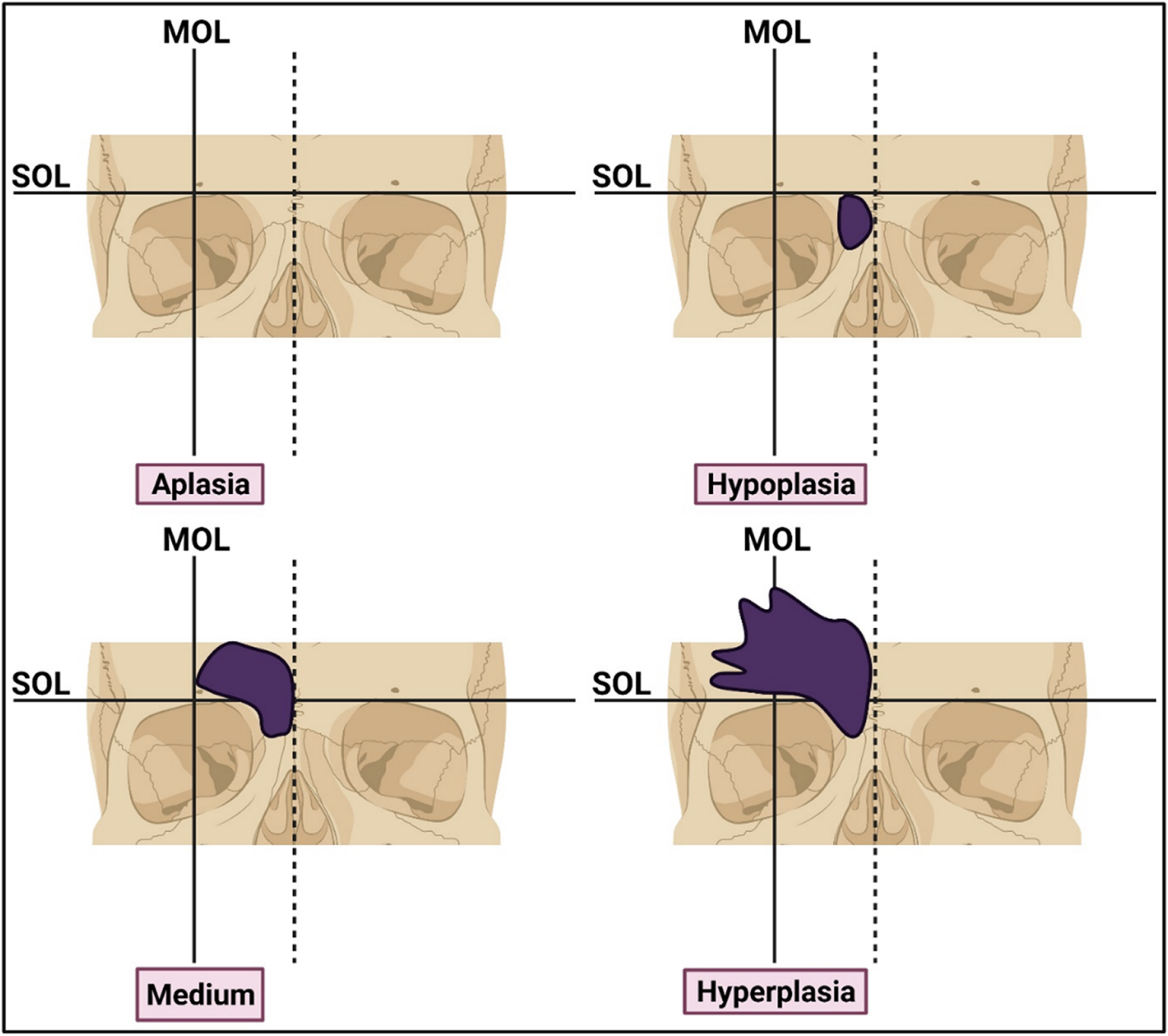
Schematic representation of morphological classification of frontal sinus size categories. SOL: supraorbital line. MOL: midorbital line. Image was prepared using biorender.com.

We evaluated right and left frontal sinuses from 610 patients’ CT scans, based on the standard method described by Guerram *et al.*, to determine the prevalence of frontal sinus size categories. ^10^ Using this method, frontal sinus size was categorized into four types, including aplasia, hypoplasia, medium-sized, and hyperplasia. To evaluate the frontal sinus categories, supraorbital and mid-orbital lines were generated on CT sections. The supraorbital line was a horizontal tangent connecting the superior margins of both orbits, while the mid-orbital line was a vertical line drawn at the midpoint of the distance between the medial and lateral borders of the orbit parallel to the mid-sagittal plane. Based on these lines, frontal sinus size categories were classified as follows: Aplasia: no pneumatization; Hypoplasia: minimal pneumatization under the supraorbital line; Medium-sized: pneumatization over the supraorbital line but medial to the mid-orbital line; Hyperplasia: lateral to the mid-orbital line ( Figure 1). All the scans were reviewed by a single observer who is a board-certified radiologist. After screening, the data from each patient was recorded in the Microsoft Excel spreadsheet.

### Statistical analysis

The statistical analysis was performed using SPSS software package (v.23) for Windows v24.0 (IBM Corp., Armonk, N.Y., USA). Descriptive statistics ( *e.g.*, frequency and percentage) were used to present the data. The sex difference was determined using the Chi-square test. A p-value <0.05 was considered statistically significant.

## Results

**Figure 2.**
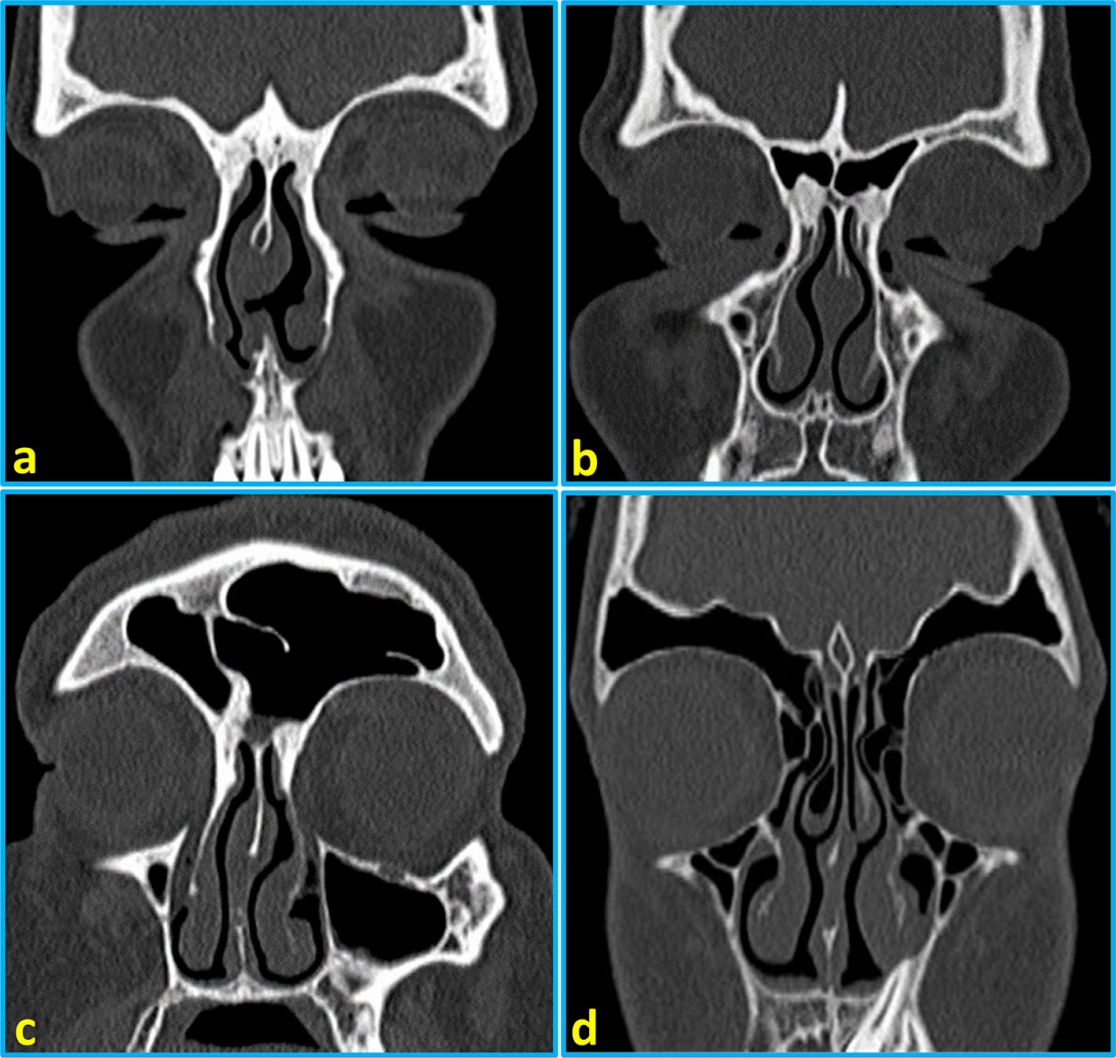
Reformatted coronal computed tomography images of the paranasal sinuses showing types of frontal sinus pneumatization: (a) bilateral aplasia of the frontal sinuses, (b) bilateral hypoplasia, (c) medium-sized pneumatization of the right frontal sinus and hyperplasia of the left frontal sinus, and (d) bilateral hyperplasia of the frontal sinuses.

In the present study, the morphometry of 1220 frontal sinuses from 610 patients was recorded concerning the frequency of each type of frontal sinus. Among the study group, 314 were males (51.5%), and 296 (48.5%) were females, with a mean age of 43.1 ± 15.5 (SD) years. The unilateral and bilateral occurrence of each type of frontal sinus frequency was summarized in Tables 1 and 2. With regard to the unilateral occurrence, the most common frontal sinus category observed was medium-sized (13.3%), followed by hyperplasia (7.9%), hypoplasia (5.4%), and aplasia (2%). Similarly, in bilateral occurrence, the most common frontal sinus category observed was medium-sized (53%), followed by hyperplasia (13.1%), hypoplasia (3.4%) and aplasia (2%) categories, respectively. Right and left frontal sinus aplasia were observed in 2.1% and 1.8% of cases, respectively. The sex-wise distribution of frontal sinus categories was presented in Tables 1 and 2. There was a significant sex difference in the frequencies of left frontal sinus hypoplasia and hyperplasia categories ( Table 1). Regarding the bilateral occurrence, a statistically significant sex difference was observed in the frequencies of hypoplasia and hyperplasia categories. The left unilateral ( *p*<0.01) and the bilateral hypoplasia ( *p*<0.05) were significantly higher in females than in males ( Tables 1 and 2). On the other hand, the left unilateral ( *p*<0.01) and the bilateral hyperplasia ( *p*<0.05) were higher in males than in females ( Tables 1 and 2). The representative images of CT scans showing frontal sinus categories are provided in Figure 2.

**Table 1.** The frequency of unilateral-frontal sinus size categories.

Frontal sinus category	Right side			Left side			Unilateral total n=1220 (%)
	Male; n=314 (%)	Female; n=296 (%)	Total n=610 (%)	Male; n=314 (%)	Female; n=296 (%)	Total n=610 (%)	
**Aplasia**	6 (1.9)	7 (2.4)	13 (2.1)	3 (1)	8 (2.7)	11 (1.8)	24 (2)
**Hypoplasia**	14 (4.5)	25 (8.4)	39 (6.4)	9 (2.9) ^*^	18 (6.1) ^*^	27 (4.4)	66 (5.4)
**Medium-sized**	50 (15.9)	37 (12.5)	87 (14.3)	39 (12.4)	36 (12.2)	75 (12.3)	162 (13.3)
**Hyperplasia**	22 (7)	13 (4.4)	35 (5.7)	41 (13.1) ^**^	20 (6.8) ^**^	61 (10)	96 (7.9)

Sex association is significant on the left side for hypoplasia and hyperplasia.

** *p*<0.01.

* *p*<0.05; Chi-square test. Values presented as number (%).

**Table 2.** The frequency of bilateral-frontal sinus size categories.

Frontal sinus category	Male n=314 (%)	Female n=296 (%)	Total n=610 (%)
**Aplasia**	5 (1.6)	7 (2.4)	12 (2)
**Hypoplasia**	5 (1.6) ^*^	16 (5.4) ^*^	21 (3.4)
**Medium-sized**	162 (51.6)	161 (54.4)	323 (53)
**Hyperplasia**	50 (15.9) ^**^	30 (10.1) ^**^	80 (13.1)

Sex association is significant for hypoplasia and hyperplasia.

** *p*<0.01.

* *p*<0.05; Chi-square test. Values presented as number (%).

## Discussion

Previously, to address the volumetric anatomic variants of the frontal sinus, three different studies have classified the frontal sinus morphology into four patterns, including aplasia or small size, hypoplasia, medium-sized, and hyperplasia. ^10^^–^^12^ These studies have used different parameters for classification with a small sample size. Moreover, these studies used dry skulls for the evaluation of variants ( Table 3). To the best of our knowledge, for the first time, the present study evaluated the frontal sinus volumetric anatomic variants in a large sample of patients in the Middle Eastern population and analyzed the gender and laterality differences in these variants. The present study followed Guerram *et al.*’s classification to determine the morphology of the frontal sinuses. ^10^ Similar to previous studies, in the present study, the medium-sized category of frontal sinus was the most common type of frontal sinus morphology. ^10^^,^^12^^,^^22^ The values of medium-sized frontal sinus frequency observed in the present study are comparable with the frequency of 65.84% reported in a recent study by Ozdemir *et al.* ^22^ In contrast, in a study by Yüksel Aslier *et al.*, hyperplasia (44.5%) was the most common type, followed by medium-sized (37.2%), hypoplasia (14.2%) and aplasia (4.1%) categories. ^11^ Similarly, in a study by Buller *et al.*, following Guerram *et al.*’s morphologic classification, hyperplasia was found to be the most frequent sinus category (66%), followed by medium-sized (30.2%) and hypoplasia (3.8%) categories. ^23^ However, no cases of aplasia were observed in this study. ^23^ The study’s small sample size and inclusion criteria could possibly be the reason for these contrasting results. After aplasia, hypoplasia of the frontal sinuses is a rare morphology of the frontal sinuses. In studies by Yuksel Aslier *et al.* and Guerram *et al.*, hypoplasia was observed in 14.2% and 9.5%, respectively. ^10^^,^^11^ The frequency of hypoplasic frontal sinuses observed in the present study (8.7%) is comparable with Guerram *et al.* study findings. ^10^ Regarding sex influence on frontal sinus categories, our study findings are comparable with the previous study by Guerram *et al.* ^10^ In their study, hypoplasia was higher in females (13.7%) than males (5%). On the other hand, hyperplasia was higher in males (16.3%) than in females (7.5%). ^10^

**Table 3.** The prevalence of frontal sinus categories in different studies.

Author, Year	Country	Sample size	Aplasia/small size (%)	Hypoplasia (%)	Medium size (%)	Hyperplasia (%)
Present study	Oman	610 patients, CT	3.9	8.8	66.2	21
Ozdemir *et al.*, 2021 ^22^	Turkey	404 patients, CT	22.9	-	65.8	11.3
Stokovic *et al.*, 2018 ^12^	Croatia	91 dry skulls, CT	21.4	-	46.7	31.9
Yuksel Aslier *et al.*, 2016 ^11^	Turkey	74 dry skulls, CT	4.1	14.2	37.2	44.5
Guerram *et al.*, 2014 ^10^	France	80 dry skulls, X-ray	2.5	9.4	76.2	11.9

Among different frontal sinus morphology variations, the frontal aplasia type is well documented in different populations worldwide. In the existing literature, the reported frequency of bilateral frontal sinus aplasia greatly varies among populations worldwide. In a recent study on Saudi individuals, bilateral frontal sinus aplasia was found to be 3.3%. ^24^ In Jordanian ^25^ and Iranian ^26^ individuals, prevalences of 4.2% and 8.3% were reported, respectively. A study from Turkey reported a low prevalence of 0.73%. ^7^ Similarly, two studies on Indian subjects reported low frequencies of 2.05% and 2.5%, respectively. ^15^^,^^27^ Contrary to these studies, high frequencies were reported in Northern Irish (10%) and Chinese individuals (16.6%). ^28^^,^^29^ Surprisingly, unusually high frequencies of 43% and 40%, respectively, in Canadian Inuit males and females, and 25% and 36%, respectively, in Native Alaskans males and females, were observed. ^30^^,^^31^ These highest frequencies were thought to be due to the influence of extremely cold climatic conditions. ^25^ In our study, the frequency of bilateral frontal sinus aplasia was noted in 2% of cases. This frequency is close to that reported in the Indian population. ^15^ Similar or comparable frequencies of frontal sinus aplasia in relative populations could be attributed to the ontogenic development of the frontal sinus. ^23^ In most studies, bilateral frontal sinus aplasia is found to be more frequent in females than in males. ^1^^,^^7^^,^^32^^,^^33^ In contrast, in Jordanian subjects, the frequency is higher in males than females. ^25^ Similar to most of the studies, bilateral frontal aplasia is found to be more common in females than in males though it was not statistically significant.

The reported frequency of unilateral frontal sinus aplasia among different populations has varied between 0.8% and 12.7%. Higher frequencies of 12.7%, 10%, 6.5%, and 6.6% unilateral aplasia were reported in Chinese, ^29^ Indian, ^15^ Saudi, ^24^ and Jordanian subjects, ^25^ respectively. In contrast, low frequencies of 1.2%, 2%, and 2.5% were reported in Turkish, ^7^ Northern Irish, ^28^ and upper Rhine subjects, ^10^ respectively. In Iranian subjects, unilateral aplasia was identified in 5.6% of cases. ^26^ In Omani subjects, the recorded unilateral aplasia frequency was low and similar to Turkish subjects. Concerning the sex differences, in Indian, ^33^ Saudi, ^24^ and Turkish subjects, ^1^ unilateral aplasia was more common in females. On the other hand, in Jordanian, ^25^ Japanese ^13^ and Iranian subjects, ^26^ unilateral aplasia was more frequent in males. In the present study, unilateral aplasia was more frequent in females than in males though it was not statistically significant. With regard to laterality differences, most of the studies from Saudi Arabia, ^24^ Japan, ^13^ Turkey, ^7^ Iran, ^26^ and India ^33^ have reported aplasia more frequently on the right side. In contrast, no laterality difference was observed in the present study.

The anatomy of the frontal sinus is the most complex compared to other paranasal sinuses. Due to its close relationship with the anterior cranial fossa and orbits, frontal sinusitis is considered a main source of orbital and cranial complications. ^34^^,^^35^ Evidence from recent studies indicates that frontal sinus anatomical variations, particularly size and shape, are positively associated with the development of sinusitis. ^16^^–^^20^ Another recent study reported a significant association between the frontal sinus type and frontal sinusitis frequency. ^22^ In the same study, the prevalence of sinusitis in medium-sized and large sinuses was significantly higher than in small sinuses. ^22^ Hence, the baseline data of frontal sinus types reported in the present study is helpful in the diagnostic evaluation of sinusitis in the clinical setting. The preoperative recognition of frontal sinus types, particularly frontal sinus aplasia in multiplanar CT scans, is crucial to avoid unexpected complications while performing endoscopic sinus surgery. For example, in endoscopic sinus surgery, opening a non-existent frontal sinus is a disastrous step. ^25^ Furthermore, frontal sinus aplasia is known to increase the risk of having traumatic brain injuries. ^36^ Hence, the frontal sinus morphology reported in the present study alert surgeons to rely on the preoperative radiological evaluation of the frontal sinus. The frontal sinus is well recognized for individual identification in forensic investigations. ^37^ The unique morphological characteristics of the frontal sinus, particularly the low prevalence of frontal sinus aplasia, are helpful in individual identification by comparing antemortem and postmortem radiographs. ^38^ Furthermore, the stability of the frontal sinus throughout life and its unique pattern, even between monozygotic twins, increased its importance in the forensic field. ^39^^,^^40^

## Conclusions

In our study, the most prevalent frontal sinus category was medium-sized, followed by hyperplasia, hypoplasia, and aplasia categories. The sex factor influenced the frequencies of hypoplasia and hyperplasia categories. The frequencies of unilateral and bilateral aplasia were low, and these values were comparable with values reported in Indian and Turkish populations. The baseline data of volumetric anatomic variations of frontal sinuses is crucial to minimize the complications associated with surgical procedures as well as for the forensic investigations.

## Data Availability

To protect the patients’ privacy the present study data access was restricted. The anonymous raw data of the study showing the different types of frontal sinus morphology can be shared with readers and reviewers. To apply for access to the data, readers or reviewers can contact Dr. Srinivasa Rao Sirasanagandla ( srinivasa@squ.edu.om). While applying for access, reader or reviewer should give a signed letter mentioning that they will not share the data with a third party and it will used only for academic purpose. The anonymous data will be provided in a password-protected file.
