# Supplementary material for: Anatomical variations of the frontal sinus: A computed tomography-based study
Source: F1000Res. 2023 Aug 31;12:71. Originally published 2023 Jan 18. [Version 2] doi: 10.12688/f1000research.129498.2 (PMC10556568; doi:10.12688/f1000research.129498.2)
Supplement: Peer review history

# Reviewer report: Reviewer response for version 2

**DOI:** 10.5256/f1000research.155087.r202735

**Published:** 2023-10-05

**Authors:** Ka Suprasanna

**Affiliations:** 1 Department of Radio-diagnosis, Kasturba Medical College, Mangalore, Manipal Academy of Higher Education, Manipal, Karnataka, India

The suggested amendments have been made. The article can be accepted

Is the work clearly and accurately presented and does it cite the current literature?

Yes

If applicable, is the statistical analysis and its interpretation appropriate?

Yes

Are all the source data underlying the results available to ensure full reproducibility?

No

Is the study design appropriate and is the work technically sound?

Yes

Are the conclusions drawn adequately supported by the results?

Yes

Are sufficient details of methods and analysis provided to allow replication by others?

Yes

Reviewer Expertise:

Neuroradiology, Head and neck Radiology

I confirm that I have read this submission and believe that I have an appropriate level of expertise to confirm that it is of an acceptable scientific standard.

# Reviewer report: Reviewer response for version 2

**DOI:** 10.5256/f1000research.155087.r202736

**Published:** 2023-09-22

**Authors:** Aspalilah Alias

**Affiliations:** 1 Department of Basic Sciences and Oral Biology, Faculty of Dentistry, Universiti Sains Islam, Nilai, Malaysia

Please kindly add ethics number in the methodology part.

Is the work clearly and accurately presented and does it cite the current literature?

Yes

If applicable, is the statistical analysis and its interpretation appropriate?

Partly

Are all the source data underlying the results available to ensure full reproducibility?

Yes

Is the study design appropriate and is the work technically sound?

Yes

Are the conclusions drawn adequately supported by the results?

Yes

Are sufficient details of methods and analysis provided to allow replication by others?

Yes

Reviewer Expertise:

Anatomist, geometric morphometric analysis, shape analysis expert, forensic anthropology, forensic odontology and radiology research.

I confirm that I have read this submission and believe that I have an appropriate level of expertise to confirm that it is of an acceptable scientific standard.

# Reviewer report: Reviewer response for version 1

**DOI:** 10.5256/f1000research.142185.r172540

**Published:** 2023-08-16

**Authors:** Ka Suprasanna

**Affiliations:** 1 Department of Radio-diagnosis, Kasturba Medical College, Mangalore, Manipal Academy of Higher Education, Manipal, Karnataka, India

The article is an interesting study regarding variations in frontal sinus pneumatisations. These are the various observations made during the review

1. Many authors have previously studied the pneumatisation of paranasal sinuses. What this study adds to the existing knowledge needs to be highlighted.
2. The authors have extensively discussed the variations in pneumatisations in various studies and in various ethnic groups. It would be more clear, if this data could be presented in a tabulated form.
3. Crista galli pneumatisation extending from the frontal sinus has been previously studied. How many of the cases in this study had crista galli pneumatisation

Is the work clearly and accurately presented and does it cite the current literature?

Yes

If applicable, is the statistical analysis and its interpretation appropriate?

Yes

Are all the source data underlying the results available to ensure full reproducibility?

No

Is the study design appropriate and is the work technically sound?

Yes

Are the conclusions drawn adequately supported by the results?

Yes

Are sufficient details of methods and analysis provided to allow replication by others?

Yes

Reviewer Expertise:

Neuroradiology, Head and neck Radiology

I confirm that I have read this submission and believe that I have an appropriate level of expertise to confirm that it is of an acceptable scientific standard, however I have significant reservations, as outlined above.

# Response

**Published:** 2023-08-24

**Authors:** Dr Srinivasa Rao Sirasanagandla

**Affiliations:** Sultan Qaboos University, Oman

Many authors have previously studied the pneumatisation of paranasal sinuses. What this study adds to the existing knowledge needs to be highlighted.

**Response:** **The importance of the study was highlighted in the discussion section.**

 The authors have extensively discussed the variations in pneumatisations in various studies and in various ethnic groups. It would be more clear, if this data could be presented in a tabulated form.

**Response:** **Table 3 summarizing the prevalence of frontal sinus variations was added in the discussion.**

 Crista galli pneumatisation extending from the frontal sinus has been previously studied. How many of the cases in this study had crista galli pneumatization.

**Response: Our study aim was to record the volumetric anatomic variants of the frontal sinus according to the Guerram *et al.*’s classification. Hence, crista galli pneumatization was not evaluated in this study.**

# Reviewer report: Reviewer response for version 1

**DOI:** 10.5256/f1000research.142185.r160660

**Published:** 2023-03-01

**Authors:** Aspalilah Alias

**Affiliations:** 1 Department of Basic Sciences and Oral Biology, Faculty of Dentistry, Universiti Sains Islam, Nilai, Malaysia

This manuscript is accepted and ready for publication after the minor changes I stated below. Overall, this finding can help in clinical cases such as sinusitis and trauma and the forensic case for identification. I hope that more statistical analysis should be done in the future, for example, the traditional morphometric methods, such as measurement of length and the volume of the frontal sinus, and more advanced analysis of the shape, such as geometric morphometric methods and artificial intelligence research for the future. The parameter for the identification, for example, age determination, can also increase the manuscript's value. Please add the ethics approval number in the methodology part as you use the patient data for the research.  

 The comments for the manuscripts as below 

1. Title - Suggested topic Anatomical variations of the frontal sinus: A computed tomography-based study. 
2. Abstract - Please change the paranasal sinus to the frontal sinus. 
3. Introduction -The introduction is enough for introducing the paper. Initially, the authors explain the anatomy of the frontal sinus, link with the previous study, and relate with the clinical and forensic function of forensic sinuses. 
4. Result: Table 1 and Table 2 Gender association is significant on the left side for hypoplasia and hyperplasia.*p<0.01.*p<0.05 
  Please change gender to sex, while *p<0.01 to **p<0.01 and maintain *p<0.05 
5. Discussion - "Two studies on Indian subjects reported low frequencies of 2.05 and 2.5%, respectively."
  Please add percentage 2.05% 
6. Discussion - Please add the forensic contribution for identification from the frontal sinus. 
7. Conclusion - Please add also the forensic contribution for identification from frontal sinus. 

Is the work clearly and accurately presented and does it cite the current literature?

Yes

If applicable, is the statistical analysis and its interpretation appropriate?

Partly

Are all the source data underlying the results available to ensure full reproducibility?

Yes

Is the study design appropriate and is the work technically sound?

Yes

Are the conclusions drawn adequately supported by the results?

Yes

Are sufficient details of methods and analysis provided to allow replication by others?

Yes

Reviewer Expertise:

Anatomist, geometric morphometric analysis, shape analysis expert, forensic anthropology, forensic odontology and radiology research.

I confirm that I have read this submission and believe that I have an appropriate level of expertise to confirm that it is of an acceptable scientific standard, however I have significant reservations, as outlined above.

# Response

**Published:** 2023-08-24

**Authors:** Dr Srinivasa Rao Sirasanagandla

**Affiliations:** Sultan Qaboos University, Oman

1. Title - Suggested topic Anatomical variations of the frontal sinus: A computed tomography-based study. 
**Response: The title of the study was changed as suggested by the reviewer.**
2. Abstract - Please change the paranasal sinus to the frontal sinus. 
**Response: The correction was made in the abstract.**
3. Introduction -The introduction is enough for introducing the paper. Initially, the authors explain the anatomy of the frontal sinus, link with the previous study, and relate with the clinical and forensic function of forensic sinuses. 
**Response: Thank you for your valuable comments**
4. Result: Table 1 and Table 2 Gender association is significant on the left side for hypoplasia and hyperplasia.*p<0.01.*p<0.05 
  Please change gender to sex, while *p<0.01 to **p<0.01 and maintain *p<0.05 
**Response: The suggested changes were made in the results section and highlighted with track changes.**
5. Discussion - "Two studies on Indian subjects reported low frequencies of 2.05 and 2.5%, respectively."
  Please add percentage 2.05% 
**Response: The correction was made.**
6. Discussion - Please add the forensic contribution for identification from the frontal sinus. 
**Response: The importance of frontal sinus for the forensic investigation was added in the discussion and highlighted with the track changes.**
7. Conclusion - Please add also the forensic contribution for identification from frontal sinus. **Response:** **The importance of frontal sinus for the forensic investigation was added in the conclusion and highlighted with the track changes.**
